# Lung Function Is Associated with Arterial Stiffness in Children

**DOI:** 10.1371/journal.pone.0026303

**Published:** 2011-10-25

**Authors:** Julian G. Ayer, Elena G. Belousova, Jason A. Harmer, Brett Toelle, David S. Celermajer, Guy B. Marks

**Affiliations:** 1 Department of Cardiology, Royal Prince Alfred Hospital, Sydney, Australia; 2 Woolcock Institute of Medical Research, Sydney, Australia; 3 Department of Medicine, University of Sydney, Sydney, Australia; 4 Heart Research Institute, Sydney, Australia; 5 The Heart Centre for Children, The Children's Hospital at Westmead, Sydney, Australia; LUMC, The Netherlands

## Abstract

**Background:**

In older adults, an independent association exists between impaired lung function and cardiovascular disease. This interaction might be related to the effects of aging and/or smoking. In order to explore possible childhood antecedents to this association, we hypothesized that decreased lung function and vascular stiffness might be related, in early life.

**Objective:**

To determine the relationship between lung function and carotid augmentation index (AIx), a measure of vascular stiffness, in 8-year old children.

**Methods:**

Data on brachial blood pressure, lung function (FEV_1_, FVC, FEV_1_/FVC, obtained by spirometry) and carotid AIx75 (AIx standardised to an arbitrary heart rate of 75 beats per minute, obtained by applanation tonometry) was available in 249 community-based 8-year old children. These healthy children had been subjects in a randomised controlled trial of two interventions (omega-3 fatty acid supplementation and house-dust mite avoidance) to prevent asthma. Smoking in pregnancy and childhood environmental tobacco smoke (ETS) exposure was prospectively collected by questionnaire. The association between lung function and carotid AIx75 was assessed in multivariate models that included sex, height, smoking status during pregnancy, ETS exposure and randomisation groups (house dust mite avoidance and dietary intervention) as covariates.

**Results:**

In the fully adjusted models, Carotid AIx75 was independently associated with FEV1 (standardised β = −0.17,b = −6.72, partial R^2^ = .02, p = 0.03), FVC (standardised β = −0.29, b = −9.31, partial R^2^ = 0.04, p<0.001) and FEV1/FVC (standardised β = .13, b = 18.4, partial R^2^ = 0.02, p = 0.04).

**Conclusion:**

Lower lung volumes are associated with increased vascular stiffness at an early age. The interaction between lung function and vascular stiffness may thus represent more than just age-related alterations in both the pulmonary and vascular systems.

## Introduction

Reduced lung function is associated with an increased incidence of cardiovascular disease, even after adjustment for the effects of traditional cardiovascular risk factors, particularly smoking [1]. Although the mechanism for this association remains largely unexplained, an association between reduced lung volumes and increased arterial stiffness has been recently described in adult populations [2], [3]. However, it is unclear whether such an association is a reflection of parallel age-related changes in pulmonary and arterial stiffness or is due to an independent interaction between arterial and pulmonary function. Thus, we examined the association between lung function and central arterial pulse wave augmentation in early childhood. Pulse wave augmentation is importantly influenced by arterial stiffness and the effects of aging and exposure to cigarette smoke are minimised in early childhood. An association present at this early age might imply a developmental or genetic basis for the association between lung function and arterial stiffness.

## Methods

### Subjects

We obtained data from a group of children who were subjects in a randomized controlled trial of interventions to prevent asthma and allergic disease. Omega-3 fatty acid dietary supplementation and house-dust mite avoidance were tested in a 2×2 factorial design from early infancy until age 5 years. Full details of the study have been previously reported [4]. Briefly, pregnant women whose unborn children had at least one parent or sibling with current asthma or wheezing were identified before birth and randomized to one of the 4 study groups. Exclusion criteria included babies from multiple births, gestational age <6 weeks, birth weight <2.5 kg, hospitalization >1 week or serious neonatal illness. Six-hundred and sixteen subjects were enrolled from September 1997 to December 1999. The children were assessed at 18 months, 3 years, 5 years (the end of the randomized intervention period) and 8 years with measurement of growth and lung function. At 8 years, the families were also invited to participate in a sub-study examining arterial structure and function.

Parents of 410 of the original children (67%) consented to participate in the vascular study. Five subjects were excluded (2 with established type 1 diabetes mellitus, 2 who consented for the study but subsequently refused cardiovascular testing and 1 who was over the pre-specified age range [8.0±0.5 years] at the time of testing) , leaving 405 participants. Carotid applanation tonometry was performed on the last 269 subjects (135 boys and 134 girls) evaluated; 6 of these were excluded with unsatisfactory carotid waveforms. Of these 263 subjects, reliable data on lung function was available in 249 and these children form the basis of this report.

### Ethics Statement

The study was approved by the Human Research Ethics Committees of the University of Sydney, the Children's Hospital at Westmead and Sydney South West Area Health Service (Western Zone). The parent or legal guardian gave written informed consent.

### Lung Function Assessment

Forced-expiratory volume in 1 second (FEV_1_), forced vital capacity (FVC) and FEV_1_/FVC ratio were measured by spirometry using a Spirocard device (QRS Diagnostic, LLC, Plymouth, MN, USA) linked to SpiroScore+ (Bird Healthcare, Melbourne, Australia) running on a laptop computer. Spirometry was performed with the child standing, wearing a nose clip and in accordance with American Thoracic Society recommendations, except that a six second expiratory time criterion was not applied. The technicians first explained and then demonstrated the procedure. The child performed the spirometric manoeuvre and the technicians corrected any errors in technique. The procedure was repeated until at least three acceptable flow volume loops had been recorded. If the best two acceptable values of FEV_1_ or the best two values of FVC were more than 5% and more than 200 ml different then the child was asked to repeat the procedure up to two more times (until the best two were within this margin). The data was reviewed post-hoc and curves that did not meet acceptability criteria were excluded. In particular, curves with evidence of cough, abrupt ending, slow start, or an extra inhalation were excluded. The highest values for FEV_1_ and FVC from any of the acceptable curves were selected for analysis. FEV_1_/FVC ratio was calculated as the ratio of these two values.

### Blood pressure and Carotid Augmentation Index

Supine left brachial blood pressure (BP) was measured twice using an automated oscillometric device (Welch Allyn Vital Signs Monitor, Skaneateles Falls, NY, USA) and the average was recorded. Augmentation Index (AIx) was measured at the left carotid artery by obtaining pulse waveforms using a high-fidelity applanation tonometer (SPC-301, Millar Instruments, Houston, Texas). Data were collected directly into a laptop computer and processed with customized waveform analysis software (Sphygmocor version 7.1, Atcor Medical, Sydney, Australia). After ten seconds of continuous pulses were recorded, an ensemble-averaged waveform was generated. This waveform was calibrated to the mean and diastolic BP; these values remain relatively constant throughout the arterial tree.

Recordings were repeated until representative averaged waveforms were obtained. Waveforms were visually inspected by a single observer, blinded to subject details, and discarded if movement and respiration artifacts were present. Data from a minimum of two averaged waveforms were used to calculate mean values that were used in subsequent analysis. AIx was calculated by the customized software as: the pressure at the 2^nd^ systolic peak–pressure at the 1^st^ systolic peak)/ pulse pressure, expressed as a percentage. Thus, AIx measures the degree of late systolic augmentation in the pulse waveform and is importantly influenced by arterial stiffness [5]. As AIx is also influenced by heart rate (HR) [6], the relationship between HR (average HR, obtained at the time of pulse wave acquisition) and AIx was determined for both sexes. The slope of the relationship (β) was utilized to standardize AIx to an arbitrary heart rate value of 75 per minute (AIx75 = AIx- β x (HR-75), β = −0.37 for boys and −0.24 for girls).

### Assessment of exposure to tobacco smoke

Prospective quantitative assessments of maternal smoking status during pregnancy, environmental tobacco smoke (ETS) exposure during the first 1 and 7 ½ year(s) of life and current (at age 8 years) ETS exposure were made by questionnaire. The details of the assessment of exposure to tobacco smoke may be found at the online supporting material (see Text S1).

### Asthma status

The prevalence of asthma was determined at age 8 years by questionnaire. Parents were asked whether their child had been ever diagnosed with asthma by a doctor or at a hospital. Use of asthma medications in the last 12 months was also determined by questionnaire. The parents and children were advised to withhold short-acting and long-acting inhaled ß-agonists for 6 and 12 hours prior to testing, respectively.

### Statistics

The association between lung function and carotid AIx75 was assessed by multivariate analysis. Likely confounding factors were based upon our previous report of arterial stiffness in this group of children [7].Standardized regression coefficients, that is, coefficients expressed in units of standard error, were reported in order to allow comparison of the strength of association between main effects measured on different scales. Statistical significance was inferred at a two-sided p-value <0.05 and analyses were performed using SAS Version 9.1.

## Results

The baseline characteristics of the 249 children in this analysis are shown in Table 1. Males and females were equally represented, as were subjects in each arm of the randomized controlled trial. Carotid AIx75 was negatively associated with FEV1 (r = −0.31, p<0.001) and FVC (r = −0.37, p<0.001) and positively associated with FEV1/FVC (r = 0.20, p = 0.002). Table 2 shows that FEV_1_, and FVC are negatively associated and FEV_1_/FVC is positively associated with carotid AIx75, after adjustment for potentially confounding factors. The full multivariate models are available in the online supporting material (see Table S1, Table S2, Table S3). The associations are independent of the influences of sex, height, smoking in pregnancy, ETS exposure and the two interventions of the randomized controlled trial. The largest effect, a negative association with FVC, indicates that lower lung volume is associated with greater carotid AIx and hence increased central arterial pulse wave augmentation. The similar, but smaller, effect of FEV_1_ is probably also related to lung volume. However, the weakly positive association with the FEV_1_/FVC ratio implies that airflow obstruction is not associated with increased central arterial pulse wave augmentation at this age.

**Table 1:** Baseline characteristics of the 249 children with data for lung function and carotid augmentation index.

Age, (Range)	7.98 (7.6–8.7)
Females, n (%)	125 (50.2%)
Diet intervention, n (%)	132 (53.0%)
HDM intervention, n (%)	121 (48.6%)
Height, cm, mean (Range)	128.3 (110.0–143.0)
Weight, kg, mean (Range)	28.8 (19.0–57.6)
BMI z-score, mean (Range)	0.48 (−2.7 to 2.61)
Prevalence of asthma, n (%)	73 (12.4%)
Asthma medication use, (%)	35 (14.1%)
Birth weight, kg, mean (Range)	3.5 (2.2–4.8)
FEV_1_, L, mean (Range)	1.53 (1.00–2.22)
FVC L, mean (Range)	1.72 (1.12–2.69)
FEV_1_/FVC, mean (Range)	0.90 (0.68–1.00)
SBP, mmHg, mean (Range)	100 (81–123)
DBP, mmHg, mean (Range)	58.5 (45–74)
Carotid AIx75, mean (Range)	−11.6 (−35.0 to 25.3)

HDM, house-dust mite; BMI, body mass index; FEV1, forced expiratory volume **(litres)** in 1 second; FVC, forced vital capacity **(litres)**; SBP, systolic blood pressure; DBP, diastolic blood pressure; AIx75, augmentation index standardized to a heart rate of 75 per minute.

**Table 2:** Association between lung function and augmentation index by multivariate analysis.

Lung Function	Carotid AIx75, %				
	Standardized estimates (β)	Raw estimates (b)	95% CIfor b	P	Partial R^2^
FEV_1_, L	−0.17	−6.72	−12.9 to −0.5	0.03	0.02
FVC, L	−0.29	−9.31	−14.7 to −4.0	<0.001	0.04
FEV_1_/FVC	0.13	18.4	0.8 to 36.1	0.04	0.02

Carotid AIx75 is the dependent variable, lung function measures are the main independent (explanatory) variables, and all models include sex, height, maternal smoking status during pregnancy, ETS exposure during childhood, and randomization groups (for house dust mite avoidance and dietary interventions) as covariates. The partial R^2^ measures the marginal contribution of that lung function measure when the other explanatory variables are already included in the multiple linear regression model and the b-coefficient represents the change in AIx75 in % per unit change in explanatory variable.

Birth weight was not significantly associated with pulse wave augmentation and entry of birth weight into the multivariate analysis had no effect on the association between lung function and carotid AIx75.

## Discussion

We have found, in healthy 8-year old children, a significant association between lower lung volumes and increased carotid pulse wave augmentation. Our finding implies that the inverse association between arterial stiffness and lung function, which has also been demonstrated in adults, may result from more than just the effects of age-related alterations in both the pulmonary and vascular systems.

A study by Bolton et al. [3] also suggested that early life developmental factors might underlie the relationship between lung function and vascular stiffness. In this study, 720 men who had lung function measured during mid-life (between 1984 and 1987 when aged 47 to 67 years) also had lung function and aortic pulse wave velocity measured in later life (between 2002 and 2004). Aortic pulse wave velocity was more strongly associated with mid-life than later life lung function. Furthermore, after mutual adjustment mid-life but not later life lung function remained significantly associated with aortic pulse wave velocity.

In a study of 157 adult subjects (mean age 67.9 years) with chronic obstructive pulmonary disease (COPD), Mcallister et al. speculated on the presence of a shared acquired or inherited tendency to develop both emphysema and increased arterial stiffness [8]. In their study, PWV had a moderate association with FEV1 % predicted (r = −0.208, p = 0.009) and FEV1/FVC (r = −0.295, p<0.001) but not with FEV1 (r = −0.012, p = 0.881). In a subset of 73 patients in whom a high resolution lung CT-scan was performed, PWV was moderately associated with the severity of emphysema (r = 0.47, p<0.001). However, potential explanatory factors such as inflammation (as measured by hs-CRP), hypoxia (as measured by the oxygen saturation) and pack-years of cigarette smoking were not significantly associated with PWV. However, in this group of adult COPD patients, the effects of early life factors were not explored. Furthermore, although an adjustment for the number of pack-years of smoking was made, there remains the possibility of residual confounding by cigarette smoking.

Our study, therefore, extend the findings of Bolton et al. and MacNee et al. by demonstrating that lower lung volumes are associated with increased carotid pulse wave augmentation from a very early age and suggesting a developmental link between lung function and arterial stiffness.

Our study has several potential limitations. Firstly, we have examined the association between lung volumes and central arterial pulse wave augmentation. Although arterial stiffness is a key determinant of pulse wave augmentation, other factors that were not measured in our study may have influenced pulse wave reflection, such as aortic diameter or the degree of aortic tapering [9]. Secondly, rather than measurement of a biomarker such as cotinine, we assessed childhood ETS exposure from questionnaires administered frequently to mothers, where information on smoking in the home was sought. Reassuringly, however, a significant proportion of the variance in early childhood cotinine levels has been shown to be due to maternal smoking, living in a home with other smokers and the number of cigarettes smoked in the home per day[10], [11], [12], [13], [14], influences which were directly assessed in our questionnaire. Furthermore, short parental questionnaires have previously been shown to accurately determine childhood ETS exposure [15]. Thirdly, the univariate associations between lung function and carotid AIx75 that we have reported are of moderate strength only and the values of partial R^2^ indicate that lung function only explains a small proportion of the variance in pulse wave augmentation as measured by carotid AIx75.

In conclusion, lower lung volumes are associated with increased pulse wave augmentation in early childhood. This finding suggests a developmental explanation for the link between lung function and arterial stiffness. Further studies are warranted to explore the factors present in early life that explain this association.

## Supporting Information

Text S1 Assessment of exposure to tobacco smoke
(DOCX)

Table S1 The association between carotid AIx75 and FEV1, after adjustment for potential confounders
(DOCX)

Table S2 The association between carotid AIx75 and FVC, after adjustment for potential confounders.
(DOCX)

Table S3 The association between carotid AIx75 and FEV1/FVC, after adjustment for potential confounders.
(DOCX)
