# Supplementary material for: Lung Function Is Associated with Arterial Stiffness in Children
Source: PLoS One. 2011 Oct 25;6(10):e26303. doi: 10.1371/journal.pone.0026303 (PMC3201952; doi:10.1371/journal.pone.0026303)
Supplement: Text S1 — Assessment of exposure to tobacco smoke (DOCX) [file pone.0026303.s001.docx]

**Exposure to tobacco smoke**

Smoking status during pregnancy was collected using a questionnaire that was completed by interview, soon after the delivery of the child. Mothers were asked “Did you smoke during the pregnancy?”. Those who answered ‘yes’ were then asked to quantify their smoking during the first, second and third trimesters as “1-10/day”, “11-20/day”, “21-40/day”, or “41+/day”. From these data, average smoking during pregnancy was calculated using mid-point values for each range (5, 15, 30 and 50, respectively). Those who denied smoking during pregnancy were classified as zero in all three trimesters.

Study nurses collected data on environmental tobacco smoke (ETS) exposure at home visits or telephone calls to home at age four weeks, then at three monthly intervals from age three months to age five years, and then at six monthly intervals to age 7 ½ years. Mothers were asked “Do you smoke?” and “Do other people living in your home smoke inside the house?” Those who answered “yes” to either of these questions were then asked to quantify, separately, the amount smoked by the mother, father, and other persons as “1-10/day”, “11-20/day”, “21-40/day”, or “41+/day”. The total number of cigarettes smoked in the house at each visit was quantified as the sum of smoking by the mother, father and others. Calculations were made by taking mid-point values for each range (5, 15, 30 and 50, respectively). Where the responses to “Do you smoke?” and “Do other people living in your home smoke inside the house?” were both “No” and there was no positive response to any of the quantitative questions about smoking, ETS exposure in the home was assumed to be zero.

Average ETS exposure was estimated over the first 12 months of post-natal life and over the first 7 ½ years of life. The mean of each consecutive pair of observations was multiplied by the interval between those consecutive observations (in months) and the sum of these means was divided by the total number of months of observation, yielding a time-weighted average for the two periods. In calculating the 12 month and 7 ½ year averages, only subjects who had data for at least six months and at least sixty months, respectively, were included.

ETS exposure was also assessed at the 8 year clinic visit, when parents were asked “Do any people living in the home where your child spends most of their time smoke inside the house?”. Those who answered ‘yes’ to this question were then asked to quantify “*cigarettes smoked inside the house*” for up to four people using the same classification as above.
