# Supplementary material for: Lung Function Is Associated with Arterial Stiffness in Children
Source: PLoS One. 2011 Oct 25;6(10):e26303. doi: 10.1371/journal.pone.0026303 (PMC3201952; doi:10.1371/journal.pone.0026303)
Supplement: Table S1 — The association between carotid AIx75 and FEV1, after adjustment for potential confounders (DOCX) [file pone.0026303.s002.docx]

Table S1: The association between carotid AIx75 and FEV1, after adjustment for potential confounders

| Variable | Standardized estimate (β) | Raw estimate (b) | 95% CI for b | P | Partial R^2^ |
| --- | --- | --- | --- | --- | --- |
| FEV1,  (Litres) | -0.17 | -6.72 | -12.9 to -0.50 | 0.03 | 0.02 |
| Sex  (male versus female) | -0.23 | -4.02 | -6.13 to -1.91 | <0.001 | 0.05 |
| Height,  (metres) | -0.13 | -19.5 | -43.2 to 4.26 | 0.107 | 0.01 |
| Smoking in pregnancy  (Yes versus No) | -0.03 | -0.68 | -4.70 to 3.33 | 0.738 | 0 |
| ETS duration in the first 12 months | -0.03 | -0.05 | -0.52 to 0.41 | 0.83 | 0 |
| ETS duration in the first 7 ½ years | 0.08 | 0.02 | -0.05 to 0.10 | 0.58 | 0 |
| HDM randomization group | 0.04 | 0.75 | -1.31 to 2.82 | 0.47 | 0 |
| Dietary randomization group | 0.07 | 1.26 | -0.82 to 3.33 | 0.23 | 0 |

FEV1, forced expiratory volume in 1 second; ETS, environmental tobacco smoke; HDM, house dust mite.
