# Supplementary material for: Lung Function Is Associated with Arterial Stiffness in Children
Source: PLoS One. 2011 Oct 25;6(10):e26303. doi: 10.1371/journal.pone.0026303 (PMC3201952; doi:10.1371/journal.pone.0026303)
Supplement: Table S2 — The association between carotid AIx75 and FVC, after adjustment for potential confounders. (DOCX) [file pone.0026303.s003.docx]

Table S2: The association between carotid AIx75 and FVC, after adjustment for potential confounders

| Variable | Standardized estimate (β) | Raw estimate (b) | 95% CI for b | P | Partial R^2^ |
| --- | --- | --- | --- | --- | --- |
| FVC,  (Litres) | -0.29 | -9.31 | -14.7 to -3.95 | <0.001 | 0.04 |
| Sex  (male versus female) | -0.19 | -3.38 | -5.51 to -1.25 | 0.002 | 0.03 |
| Height,  (Metres) | -0.05 | -7.35 | -31.6 to 16.9 | 0.55 | 0 |
| Smoking in pregnancy  (Yes versus No) | -0.05 | -1.02 | -4.98 to 2.94 | 0.61 | 0 |
| ETS duration in the first 12 months | -0.06 | -0.10 | -0.57 to 0.36 | 0.66 | 0 |
| ETS duration in the first 7 ½ years | 0.11 | 0.03 | -0.05 to 0.10 | 0.44 | 0 |
| HDM randomization group | 0.05 | 0.85 | -1.19 to 2.88 | 0.41 | 0 |
| Dietary randomization group | 0.07 | 1.16 | -0.88 to 3.20 | 0.26 | 0 |

FEV1, forced expiratory volume in 1 second; ETS, environmental tobacco smoke; HDM, house dust mite.
