# Supplementary material for: Lung Function Is Associated with Arterial Stiffness in Children
Source: PLoS One. 2011 Oct 25;6(10):e26303. doi: 10.1371/journal.pone.0026303 (PMC3201952; doi:10.1371/journal.pone.0026303)
Supplement: Table S3 — The association between carotid AIx75 and FEV1/FVC, after adjustment for potential confounders. (DOCX) [file pone.0026303.s004.docx]

Table S3: The association between carotid AIx75 and FEV1/FVC, after adjustment for potential confounders.

| Variable | Standardized estimate (β) | Raw estimate (b) | 95% CI for b | P | Partial R^2^ |
| --- | --- | --- | --- | --- | --- |
| FEV1/FVC,  (Litres) | 0.13 | 18.4 | 0.75 to 36.1 | 0.04 | 0.02 |
| Sex  (male versus female) | -0.23 | -4.09 | -6.19 to -1.99 | <0.001 | 0.05 |
| Height,  (Metres) | -0.22 | -33.6 | -51.7 to -15.4 | 0.0003 | 0.05 |
| Smoking in pregnancy  (Yes versus No) | -0.05 | -1.06 | -5.09 to 2.97 | 0.61 | 0 |
| ETS duration in the first 12 months | -0.06 | -0.11 | -0.58 to 0.36 | 0.65 | 0 |
| ETS duration in the first 7 ½ years | 0.12 | 0.03 | -0.04 to 0.11 | 0.12 | 0 |
| HDM randomization group | 0.06 | 1.04 | -1.03 to 3.11 | 0.32 | 0 |
| Dietary randomization group | 0.06 | 0.99 | -1.08 to 3.07 | 0.35 | 0 |

FEV1, forced expiratory volume in 1 second; ETS, environmental tobacco smoke; HDM, house dust mite.
